# Case Report: Whole Exome Sequencing Identifies Compound Heterozygous Variants in *TSFM* Gene Causing Juvenile Hypertrophic Cardiomyopathy

**DOI:** 10.3389/fcvm.2021.798985

**Published:** 2022-01-06

**Authors:** Jamie O. Yang, Hapet Shaybekyan, Yan Zhao, Xuedong Kang, Gregory A. Fishbein, Negar Khanlou, Juan C. Alejos, Nancy Halnon, Gary Satou, Reshma Biniwale, Hane Lee, Glen Van Arsdell, Stanley F. Nelson, Marlin Touma, the UCLA Clinical Genomics Center, the UCLA Congenital Heart Defects-BioCore Faculty

**Affiliations:** ^1^David Geffen School of Medicine, University of California, Los Angeles, Los Angeles, CA, United States; ^2^Department of Pediatrics, David Geffen School of Medicine, University of California, Los Angeles, Los Angeles, CA, United States; ^3^Neonatal/Congenital Heart Laboratory, Cardiovascular Research Laboratory, University of California, Los Angeles, Los Angeles, CA, United States; ^4^Department of Pathology and Laboratory Medicine, David Geffen School of Medicine, University of California, Los Angeles, Los Angeles, CA, United States; ^5^Department of Human Genetics, David Geffen School of Medicine, University of California, Los Angeles, Los Angeles, CA, United States; ^6^Institute for Precision Health, David Geffen School of Medicine, University of California, Los Angeles, Los Angeles, CA, United States; ^7^Department of Pediatrics, David Geffen School of Medicine, Children's Discovery and Innovation Institute, University of California, Los Angeles, Los Angeles, CA, United States; ^8^The Molecular Biology Institute, David Geffen School of Medicine, University of California, Los Angeles, Los Angeles, CA, United States; ^9^Eli and Edythe Broad Stem Cell Research Center, David Geffen School of Medicine, University of California, Los Angeles, Los Angeles, CA, United States

**Keywords:** mitochondrial cardiomyopathy, hypertrophic cardiomyopathy, COXPD3, whole exome sequencing, mitochondrial hyperplasia, TSFM

## Abstract

We report a case of hypertrophic cardiomyopathy and lactic acidosis in a 3-year-old female. Cardiac and skeletal muscles biopsies exhibited mitochondrial hyperplasia with decreased complex IV activity. Whole exome sequencing identified compound heterozygous variants, p.Arg333Trp and p.Val119Leu, in *TSFM*, a nuclear gene that encodes a mitochondrial translation elongation factor, resulting in impaired oxidative phosphorylation and juvenile hypertrophic cardiomyopathy.

## Introduction

Inherited mitochondrial diseases, or diseases leading to a defect in mitochondrial oxidative phosphorylation, are estimated to occur in 1 in 5,000 live births (1). Mitochondrial diseases are uniquely under dual control by the maternally inherited mitochondrial genome and the Mendelian-inherited nuclear genome. There is a total of 37 genes in the mitochondrial genome coding for rRNAs, tRNAs, and subunits of the respiratory chain. In contrast, there are over 900 nuclear gene products that code for mitochondrial component. In adults, the vast majority of identified mitochondrial respiratory chain defects are associated with mitochondrial DNA (2). In children however, pathogenic variants in mitochondrial DNA are estimated to account for <10% of all mitochondrial disorders, with nuclear DNA defects being the main cause of mitochondrial disease (2). With the advent of next generation sequencing platforms, including whole exome sequencing (WES), nuclear DNA-encoded genes are increasingly recognized to also cause mitochondrial respiratory chain defects. A growing number of disease-causing variants in nuclear genes are being identified including elongation factors (EF-Ts), mitoribosomal proteins, aminoacyl tRNA synthetases, and release factors.

Mitochondrial diseases present heterogenously affecting multiple organ systems with variable clinical severity, but they particularly affect organs with high metabolic demand such as the heart due to the crucial role of mitochondria in energy production. One of the most common cardiac manifestations is cardiomyopathy, occurring in 20–40% of children with mitochondrial diseases (3). Primary mitochondrial cardiomyopathy is defined as abnormal myocardial structure or function caused by genetic defects involving the mitochondrial respiratory chain without the presence of coronary artery disease, hypertension, valve disease, or congenital heart disease. The clinical phenotype of mitochondrial cardiomyopathy is highly variable. In the pediatric population, inborn errors of metabolism account for <10% of pediatric cardiomyopathy, with a large proportion of these cases being primarily due to mitochondrial oxidative phosphorylation defects (4). The most commonly affected nuclear and mitochondrial encoded genes with pathogenic variants causing pediatric mitochondrial cardiomyopathies are systematically reviewed by Enns et al. (2).

Herein, we report a unique case of primary mitochondrial cardiomyopathy in a female toddler who presented with cardiomegaly and advanced left ventricular failure at 3 years of age. Blood lactate, lactate-to-pyruvate ratio, brain type-natriuretic peptide (BNP) and cardiac troponin levels were elevated. Biopsies from cardiac and skeletal muscles exhibited mitochondrial hyperplasia of abnormal morphology and diminished complex IV activity. Targeted sequencing of mitochondrial encoded genes was negative. Trio (proband and parents) WES revealed novel compound heterozygous variants in *TSFM* [NM_001172696.2; OMIM 604723], a nuclear gene encoding a mitochondrial translation elongation factor (EF-T), that resulted in impaired mitochondrial biogenesis and respiratory function, leading to early-onset hypertrophic cardiomyopathy. Highlighted in this report are the unique pathological and molecular features of this extremely rare mitochondrial cardiomyopathy.

### History of Presentation

**Figure 1:**
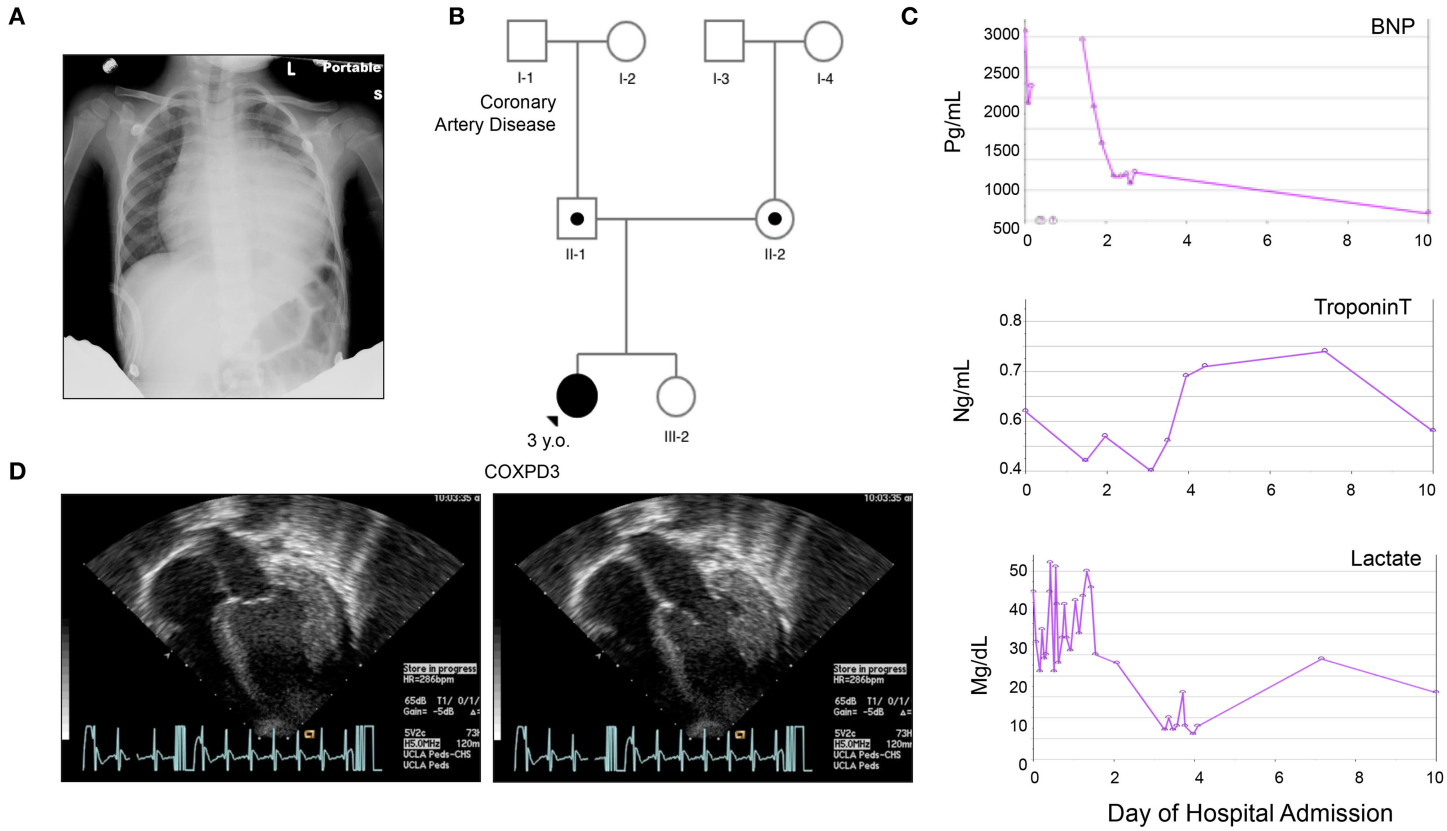
Clinical Presentation of the Proband. **(A)** Chest X ray on admission demonstrating severe cardiomegaly. **(B)** Three-generation family pedigree: III-1, Index case; II-1, father carries the c.355G>C (p.Val119Leu) variant; II-2, mother carries the c.997C>T (p.Arg333Trp) variant. III-2, older healthy sister that has not been tested; No other family history of heart disease besides [I-1], grandfather with coronary heart disease. **(C)** Graphs representing summary of serum brain-natriuretic peptide (BNP), cardiac Troponin T, and Lactate levels during the first 10 h after admission. **(D)** Representative echocardiogram, apical views on admission during systole (a) and diastole (b) demonstrating global left ventricular hypokinesis with estimated EF of 10–15%, significant concentric hypertrophy of the left ventricle, normal coronary arteries take offs, circumferential effusion without chamber compression measuring 14 mm posterior and 8 mm anterior, mild mitral regurgitation. *Please see accompanied Supplementary Videos included in Supplementary Materials.

The proband is a previously healthy 3-year-old female of non-consanguineous Caucasian parents, who presented with a 10-day history of cough, rhinorrhea, fever, and diarrhea and a 2-day history of emesis, found to have severe cardiomegaly on X-ray (Figure 1A). She had no prior history of shortness of breath, syncope or chest pain. She was born at term without complications after an uneventful pregnancy. Early growth and development were typical (able to walk by 14 months) and she reported no difficulty with running and keeping up with peers. Her older sister and both parents were healthy. The family history was significant for a paternal grandfather with adult-onset coronary artery disease, but otherwise non-contributory. There was no history of congenital disease or sudden infant deaths in the family. Physical examination was largely unremarkable. Three generation-family pedigree is shown in Figure 1B.

### Investigations

#### Clinical Workup

On admission, a chest X-ray demonstrated profound cardiomegaly (Figure 1A). Her BNP, cardiac troponin, and serum lactate were elevated (Figure 1C). She was found to have severe dilated cardiomyopathy with concentric left ventricular thickening and an ejection fraction (EF) of 10-15% per Echocardiography (Figure 1D and Supplementary Video). Her EKG was profoundly abnormal with huge voltages suggesting a storage disease (Supplementary Figure 1). Subsequently, a cardiac catheterization was done, which demonstrated elevated filling pressures in both ventricles and increased systemic vascular resistance, but normal caliber coronary arteries, aorta, and renal arteries, effectively ruling out renal artery stenosis and aortic coarctation as causes of the cardiomyopathy. Infectious workup for myocarditis also came back negative. Metabolic workup revealed metabolic acidosis (PH 7.3), mildly elevated blood lactate level of 2.94 nmol/L (norm: 0.56–1.39) with a lactate/pyruvate ratio of 18.375 (pyruvate: 0.16 nmol/L, norm: 0.03–0.2).

#### Biopsy Findings

**Figure 2:**
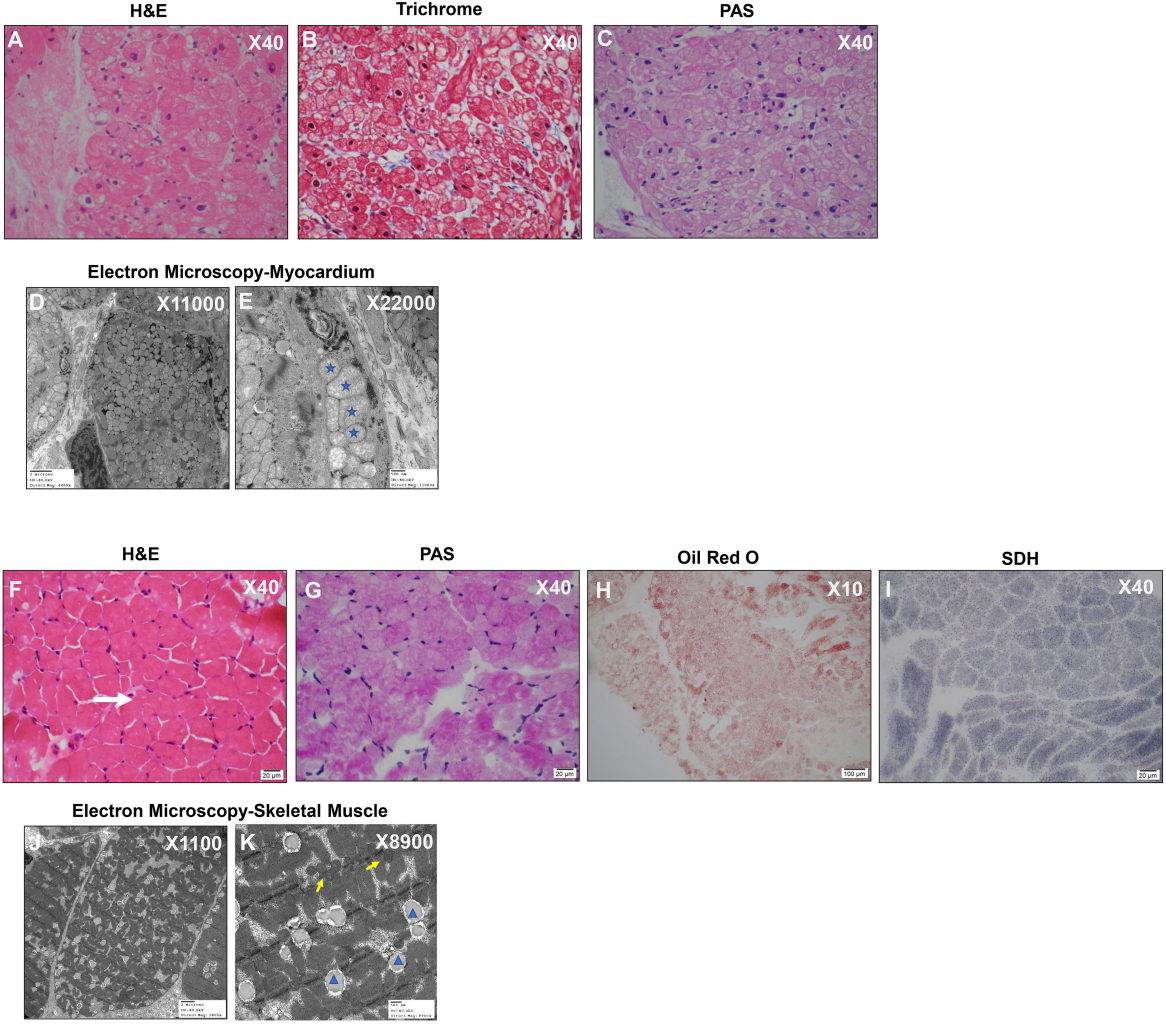
Myocardium and Skeletal Muscle Biopsies Reveal Hyperplastic and Polymorphic Mitochondria and Lipid Accumulation. **(A)** Myocardium: H&E X40 shows diffuse generalized granular vacuolization likely representing mitochondria. **(B)** Myocardium: Mason Trichrome X40 reveals minimal fibrosis. **(C)** Myocardium: Periodic Acid Schiff (PAS) X40 Negative staining indicates absence of abnormal glycogen content. **(D,E)** Myocardium: Electron microscopy– myocardium X11000 **(D)** and X22000 **(E)**: Mitochondrial hyperplasia, pleoconia, and megaconial forms and loss of internal cristae; Stars indicate mitochondria of variable and abnormal size. **(F)** Skeletal Muscle: H&E X40 Moderate fiber size variation with occasional atrophic fibers (white arrow). No evidence of degeneration/regeneration/inflammation. **(G)** Skeletal Muscle: PAS X40 Mild glycogen accumulation. **(H)** Skeletal Muscle: Oil Red O X10 Increased cytoplasmic lipid. **(I)** Skeletal Muscle: SDH X40 complex II mitochondrial respiratory chain enzyme-normal appearance. **(J,K)** Electron microscopy-skeletal muscle direct magnification of X11000 **(J)** and X8900 **(K)**: Mitochondria are normal in size and shape (yellow arrows); there is slight increase in cytoplasmic Glycogen (orange star) lipid (blue arrowhead).

An endomyocardial biopsy taken at the time of the catheterization revealed prominent vacuolar cardiomyocytes with finely granular component in hypertrophied myofibers, likely representing mitochondrial hyperplasia. Periodic Acid Schiff (PAS) staining of the cardiac specimen revealed no glycogen storage abnormalities, and no evidence of inflammation or necrosis was detected (Figures 2A–C). At the ultrastructural level, electron microscopic images revealed inter-myofibrillar mitochondrial hyperplasia with polymorphic features including pelaconia and megaconia forms. Relative paucity of internal cristae was observed, and linear structures representing mitochondrial DNA were noted, together, raising suspicion for a primary mitochondrial disorder (Figures 2D,E).

Two skeletal muscle biopsies from the thigh (quadriceps muscle) were subsequently obtained to assess for global myopathy. The skeletal muscle showed evidence of lipid and glycogen accumulation suggestive of a combined lipid and glycogen storage myopathy and there was T1 myofiber predominance with preferential type 2 myofiber atrophy (Figures 2F–H). A primary carnitine deficiency or glycogen storage disorder was suspected. However, plasma free/total acyl-carnitine profile was normal and analysis of exons 2-20 of the glucosidase alpha acid (*GAA*) gene from her blood revealed no mutation known to be associated with Glycogen Storage Disease II/Pompe disease (Supplementary Table 1). No mitochondrial abnormality was detected by immunohistochemistry (Figure 2I), and electron microscopy analysis of skeletal myotubes was essentially normal (Figures 2J,K). Hence, biochemical analysis of mitochondrial electron transport complexes was performed on skeletal muscle specimen homogenate and revealed significant reduction in COX activity (16% of the controls) consistent with deficient electron transport complex IV deficiency.

#### Genetic Workup

The phenotype of mitochondrial IV deficiency can be contributed by disease-causing variants in the mitochondrial genome or in the nuclear mitochondrial genes. To identify the potential genetic defect responsible for the observed cardiac phenotype, a targeted sequence analysis of the mitochondrial DNA coding genes for COX (Complex IV) subunits, *COI, COII*, and *COIII* was first performed but no known pathogenic variants were identified. Likewise, the nuclear genes associated with complex IV including *COX10, COX15, COX6B1, SCO1, SCO2, SURF1*, and *TACO1*, were sequenced, and no significant variations were detected. Sequencing of *FASTKD2*, a nuclear gene that is involved with mitochondrial RNA maturation, was also negative for deleterious variants.

#### Whole Exome Sequencing

**Figure 3:**
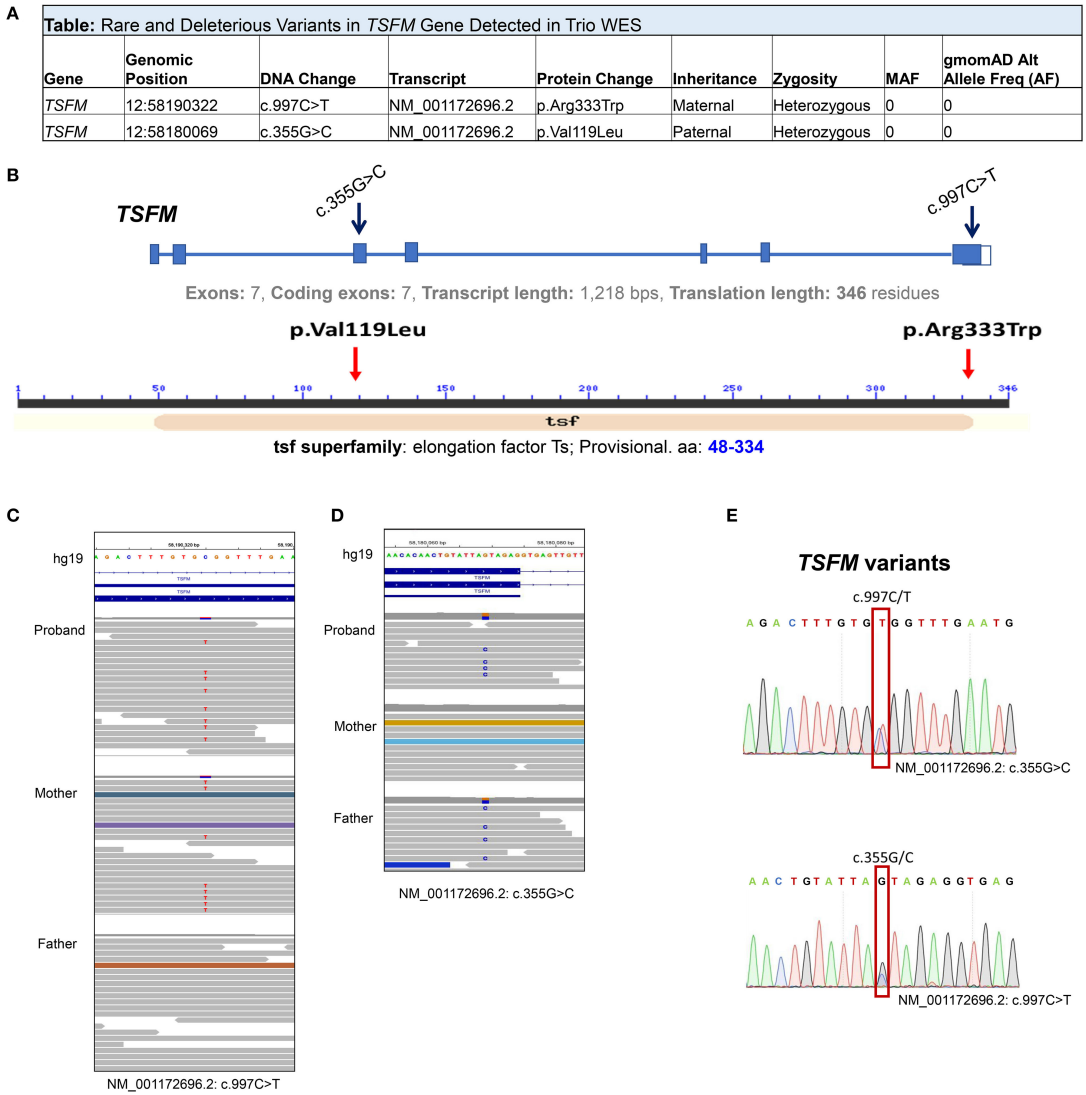
Compound Heterozygous Variants of *TSFM* Revealed by Trio (Proband/Parents) Whole Exome Sequencing (WES). **(A)** Table summary depicts rare compound heterozygous variants in *TSFM* genes detected by WES. **(B)** Schematic representation of *TSFM* gene (upper) and TSFM protein (lower) and known structural motifs. Black arrows indicate exons affected by deleterious mutations. Red arrows indicate the positions of AA substitutions in TSFM protein sequence caused by *TSFM* variants. **(C,D)** Integrated genomic viewer windows depict tow heterozygous variants in *TSFM* in the proband genome, the maternally inherited c.997C>T **(C)** and the paternally inherited c.355G>C **(D)**. **(E)** Sanger sequencing confirms that both *TSFM* variants are in trans configuration in the proband DNA.

Trio WES was subsequently performed on the proband and both parents. No regions of homozygosity [>5 MB] were identified in the proband's genome suggesting there is no evidence of consanguinity, large deletions or uniparental disomy. Two novel compound heterozygous variants in the *TSFM* gene (NM_001172696.1) were detected in trans: c.997C>T [p.(Arg333Trp)] and c.355G>C [p.(Val119Leu)] (Figures 3A–D). Both variants were confirmed in the proband DNA through Sanger sequencing (Figure 3E). Pathogenic Variants in *TSFM* are known causal for autosomal recessive combined oxidative phosphorylation deficiency type 3 syndrome (COXPD3) [OMIM: 610505] with heterogenous clinical presentation including cardiomyopathy, encephalopathy, leigh disease, ataxia, or combinations of these disorders. *TSFM* encodes the mitochondrial translation elongation factor Ts. The encoded protein (EF-Ts, 346 aa) is an enzyme that catalyzes the exchange of guanine nucleotides on the translation elongation factor Tu during the elongation step of mitochondrial protein translation, thereby, brings an amino-acylated tRNAs to the ribosomal A site as a ternary complex with guanosine triphosphate (GTP).

The maternally inherited variant c.997C>T is predicted to be a p.(Arg333Trp) change in the EF-Ts subdomain interacting with EF-Tu and has been previously reported as pathogenic (5–7) (dbSNP; rs121909485) and disease-causing for COXPD3 (ClinVar ID: 5379). The paternally inherited c.355G>C is predicted to be a p.(Val119Leu) change. This variant was not previously reported in literature or Clin Var, and was annotated as a variant of uncertain significance (VUS) in Varsome https://varsome.com/variant/hg38/rs863224936?annotation-mode=germline. This variant is not observed in the normal population database, gnomAD, and is highly evolutionally conserved. Substitution of Valine with Leucine is predicted to be deleterious/probably damaging by three *in silico* prediction algorithms (SIFT, PolyPhen2, Condel). Being in trans with a known pathogenic variant in a known autosomal recessive disease that fits the phenotype of the patient well, the variant is classified as likely pathogenic according to published American College of Medical Genetics and Genomics (ACMGG) standards and guidelines and this compound heterozygous genotype is considered diagnostic.

### Management

**Figure 4:**
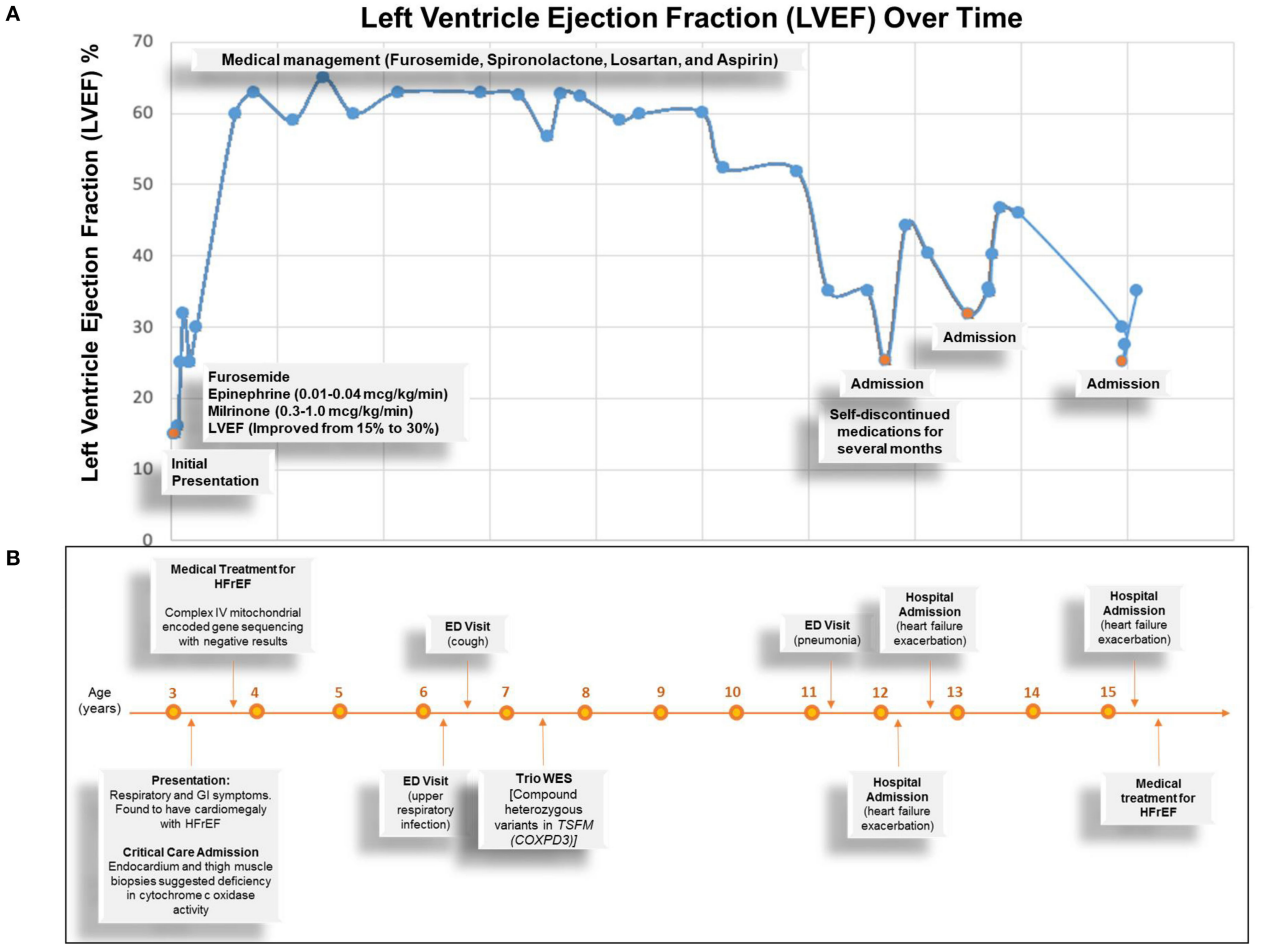
Summary of Clinical Time Course of the Proband. **(A)** Graph depicts left ventricle ejection fraction percentage overtime. Blue dots represent the time of measurements. Orange dots represent the times of admission and related events (texts). **(B)** Summary of clinical time course depicts important clinical events over time. HFrEF, heart failure with reduced ejection fraction; ED, emergency department; COXPD3, combined oxidative phosphorylation deficiency 3.

On admission, she was treated with continuous intravenous (IV) infusion of milrinone (0.3-1.0 mcg/kg/min) for ionotropic support and afterload reduction, IV furosemide for diuresis, and IV heparin for anticoagulation. The patient was also initially given continuous IV infusion of epinephrine (0.01-0.04 mcg/kg/min) for 3 days, which was subsequently discontinued after lactate downtrend, and later avoided due to concern for its effect in increasing afterload. With remarkable improvement of ejection fraction from 10 to 30% and alleviation of heart failure symptoms, she was discharged with oral therapy of lisinopril, furosemide, chlorothiazide, spironolactone, and aspirin. A summary of clinical time-course and cardiac function, represented by percentage left ventricle ejection fraction (LVEF%), is shown in Figure 4.

At the time of this publication, the patient is 15 years old with mild symptoms of heart failure [New York Heart Association (NYHA) class II-III] and left ventricular ejection fraction >35%, but progressively worsening exercise tolerance with a maximum rate of oxygen (VO2max) of 11 ml/kg. She has a low anaerobic threshold and is not physically active though attends school and participates in usual activities of daily living. She has been hospitalized four times for acute heart failure exacerbations since her initial presentation, which typically resolve within a few days with aggressive diuresis. She was evaluated for cardiac transplantation at the time of presentation. However, she did not qualify because her heart failure symptoms stabilized on oral medications. Her chronic cardiomyopathy therapy includes aspirin, furosemide, losartan, and spironolactone. Her parents and older sister remain asymptomatic.

To date, it appears that the primary clinical manifestations and pathological findings of COXPD3 are restricted to the heart. The skeletal muscles, while exhibiting biochemical features of the disorder, seem to have been spared with no other notable organ involvement. The patient has not manifested signs of muscle weakness, though anaerobic threshold is low. Her neurological, neurosensory and executive functions remain normal.

## Discussions

In this report, we describe an isolated concentric hypertrophic cardiomyopathy in a 3-year-old proband carrying compound heterozygous variants in *TSFM*, the c.997C>T [p.(Arg333Trp)] variant and the c.355G>C [p.(Val119Leu)] variant, both in highly conserved regions of the TSFM protein. We present the unique histopathological characteristics and the genetic findings of this rare early-onset mitochondrial cardiomyopathy.

*TSFM* encodes a mitochondrial translation elongation factor Ts (EF-Ts). The encoded protein (346 aa) is a guanine nucleotide exchange factor that plays an essential function during the elongation step of mitochondrial protein translation (8). Pathogenic variants in *TSFM* are known causal for COXPD3 with variable presentations ranging from fatal neonatal-onset to moderate disease courses.

Disease causing variants in *TSFM* are extremely rare. To date, disorders caused by variants in the *TSFM* gene have been reported in only 17 patients and published in 11 reports. These reports are summarized in Supplementary Table 2. In terms of cardiomyopathy, variable clinical phenotypes have been correlated with *TSFM* pathogenic variants, including hypertrophic cardiomyopathy, dilated cardiomyopathy, arrhythmogenic cardiomyopathy with fibro-adipose replacement and biventricular failure. These cases were isolated, or associated with varying levels of multisystem involvement (Supplementary Table 2).

The p.(Arg333Trp) variant identified in our proband, has been previously detected in seven patients, transmitted in an autosomal recessive manner, manifesting with heterogeneous phenotypes and are all associated with death during infancy. Smeitink et al. reported two unrelated patients with this variant, one with mitochondrial encephalomyopathy, and another patient with concentric hypertrophic cardiomyopathy and muscular hypotonia, both of whom died at 7 weeks (5). Vedrenne et al. reported the same *TSFM* variant in two patients with infantile liver failure (6). Two other patients with this homozygous variant had cardioencephalomyopathy and COXPD3 (7).

The p.(Val119Leu) variant is a likely pathogenic heterozygous variant occurring at a highly conserved residue within the protein sequence, in the setting of a compound *TSFM* variant. Furthermore, this variant is associated with a pathogenic variant in a known autosomal recessive disease, in which the phenotypic, the histopathological findings, and the impaired mitochondrial complex IV activity are consistent with mitochondrial disease caused by pathogenic variants in the *TSFM* gene. Remarkably, no cases with homozygous *TSFM* [p.(Val119Leu)] genotype have been reported to date.

There are several important points to be highlighted in this report. Firstly, our patient's phenotype supports that EF-Ts and mitochondrial translation are critical for cardiac function in early childhood. It is estimated that about 20–30% of children with mitochondrial disease will develop hypertrophic cardiomyopathy (3). Patients with mitochondrial translation defects often present with cardiomyopathy at a young age (3); however, as seen in our patient, if patients can survive during their initial presentation, their cardiomyopathy may potentially remain fairly stable without needing a heart transplant into their adolescent years. This highlights the importance of early diagnosis and aggressive treatment to support cardiac function in the early stages, and the value of developing clinical algorithms for mitochondrial disease-related cardiomyopathy. Understanding the natural history and its correlation with genotypic information may inform treatment decisions and prioritization for heart transplantation.

Secondly, the phenotypes previously described in patients with homozygous genotype for the recurrent p.(Arg333Trp) variant caused infantile deaths, whereas our patient with a compound heterozygous variant survived infancy with a stable cardiomyopathy. Previously described compound heterozygous variants in *TSFM* also demonstrated improved survival with patients living into their adolescent years (8). This suggests that although the number of reported cases is not sufficient to establish a phenotype-genotype correlation, compound heterozygous mutations in *TSFM* appear less severe, as the severe juvenile cardiac phenotype stabilized later. The mechanism of this stabilization remains to be fully revealed, but potentially due to mitochondrial hyperplasia and residual functional EF-Ts allowing for mitochondrial translation to occur, or secondary to compensatory upregulation in EF-Tu that was demonstrated to rescue EF-Ts deficits.

Thirdly, as seen in the previously published reports of *TSFM* variants, phenotypes can range from mitochondrial encephalopathy to optic atrophy, Leigh syndrome, and cardiomyopathy. The reasons that underlie the high variability of phenotypes are unclear at this time (9). Tissue specific genetic modifiers likely exist, but their identification is a challenge given the rarity of these variants (5). This also leads to difficulties with providing accurate prognosis to patients and their families. From the current published *TSFM* cases, hypertrophic cardiomyopathy is the most common manifestation, occurring in 65% of patients. This is followed by hypotonia (41% of patients) and optic atrophy (29% of patients). Recently, *TSFM* has also been linked to hyperkinetic movement disorders. Continued monitoring of these patients as well as genetic screening and employing WES for early detection of new patients with similar phenotypes will be invaluable in furthering our understanding of mitochondrial oxidation phosphorylation deficiency diseases and informing clinical care.

Lastly, based on the currently reported cases, more than 75% of patients with pathogenic *TSFM* variants have lactic acidosis on presentation. In one *TSFM* case, treatment with sodium dichloroacetate (DCA), an activator of pyruvate dehydrogenase, helped decrease left ventricle mass in a patient with hypertrophic cardiomyopathy (10). Although there is currently no targeted therapy for *TSFM* gene variants, lowering lactate levels with DCA, in combination with L-carnitine and Coenzyme Q supplementation, may potentially lend therapeutic value in the future.

## Conclusions

Pathogenic variants of *TSFM* gene present heterogeneously, and presentation and prognosis can be more severe in homozygous recessive cases. Our report implicates novel compound heterozygous variants, extends the morphologic phenotypes associated with *TSFM* mutations, and confirms that the heart is the main target. EF-Ts and mitochondrial translation apparatus are critical for cardiac function in early childhood, and if the patient survives the critical years, the cardiac status may stabilize. Isolated juvenile cardiomyopathy should prompt consideration of primary mitochondrial dysfunction caused by potential mutations in either mitochondrial or nuclear mitochondrial genes involved in the mitochondrial oxidative phosphorylation process. Whole exome sequencing can improve early detection of causal nuclear mitochondrial genes and facilitate early diagnosis, genetic counseling and discovery of novel treatment strategies.

## Data Availability Statement

The data presented in this study must be deposited and made publicly available in an acceptable repository, prior to publication. The study was registered in dbGaP under Novel Gene-Environment Regulatory Circuit in Chamber-Specific Growth of Perinatal Heart, Study ID: 45333. The stable dbGaP accession for this study is phs002725.v1.p1.

## Ethics Statement

The studies involving human participants were reviewed and approved by University of California Los Angeles-Institutional Review Board. Written informed consent to participate in this study was provided by the participants' legal guardian/next of kin.

## Author Contributions

JY collected the data, participated in data analysis, and drafted the manuscript. HS, XK, and YZ participated in data analysis and manuscript writing. GF and NK performed pathological studies. NH, GS, and JA participated in patient enrollment and provided clinical insights. RB and GVA provided tissue specimens. HL and SN participated in genetic studies. MT conceived and designed the project, analyzed most of the data, obtained funding, and edited the manuscript. All authors contributed to the article and approved the submitted version.

## Funding

This work was supported by grants from NIH/NHLBI 1R01 HL153853-01 (MT), the Department of Defense-Congressionally Directed Medical Research Programs W81XWH-18-1-0164 (MT), and the UCLA Center for Clinical Translational Science Institute Research Grant NIH/UL1TR000124 (MT).

## Conflict of Interest

HL is now employed by 3billion, Inc. but her contribution to the manuscript was completed while she was employed at UCLA. The remaining authors declare that the research was conducted in the absence of any commercial or financial relationships that could be construed as a potential conflict of interest.

## Publisher's Note

All claims expressed in this article are solely those of the authors and do not necessarily represent those of their affiliated organizations, or those of the publisher, the editors and the reviewers. Any product that may be evaluated in this article, or claim that may be made by its manufacturer, is not guaranteed or endorsed by the publisher.

## Abbreviations

WES: Whole exome sequencing
EFTs: Elongation Factor Ts
EFTu: Elongation Factor Tu
PAS: Periodic acid-Schiff stain
MAF: Minor Allele Frequency
COXPD3: Combined oxidative phosphorylation deficiency 3
DCA: Dichloroacetate.

## References

[1] Koenig MK. Presentation and diagnosis of mitochondrial disorders in children. Pediatr Neurol. (2008) 38:305–13. 10.1016/j.pediatrneurol.2007.12.001 18410845 PMC3099432

[2] Enns GM. Pediatric mitochondrial diseases and the heart. Curr Opin Pediatrics. (2017) 29:541–51. 10.1097/MOP.0000000000000535 28719387

[3] Holmgren D Wåhlander H Eriksson BO Oldfors A Holme E Tulinius M. Cardiomyopathy in children with mitochondrial disease; clinical course and cardiological findings. Eur Heart J. (2003). 24:280–8. 10.1016/S0195-668X(02)00387-1 12590906

[4] Linhart A. Metabolic Cardiomyopathy. In: Clinical Cardiogenetics, editors. Cham: Springer International Publishing (2020). p. 151–66. 10.1007/978-3-030-45457-9_9

[5] Smeitink JA Elpeleg O Antonicka H Diepstra H Saada A Smits P. Distinct clinical phenotypes associated with a mutation in the mitochondrial translation elongation factor EFTs. Am J Hum Genet. (2006) 79:869–77. 10.1086/508434 17033963 PMC1698578

[6] Vedrenne V Galmiche L Chretien D de Lonlay P Munnich A Rötig A. Mutation in the mitochondrial translation elongation factor EFTs results in severe infantile liver failure. J Hepatol. (2012) 56:294–7. 10.1016/j.jhep.2011.06.014 21741925

[7] Calvo SE Compton AG Hershman SG Lim SC Lieber DS Tucker EJ. Molecular diagnosis of infantile mitochondrial disease with targeted next-generation sequencing. Sci Transl Med. (2012) 4:118ra10. 10.1126/scitranslmed.3003310 22277967 PMC3523805

[8] Ahola S Isohanni P Euro L Brilhante V Palotie A Pihko H. Mitochondrial EFTs defects in juvenile-onset Leigh disease, ataxia, neuropathy, optic atrophy. Neurology. (2014) 83:743–51. 10.1212/WNL.0000000000000716 25037205 PMC4150129

[9] Pickett SJ Grady JP Ng YS Gorman GS Schaefer AM Wilson IJ. Phenotypic heterogeneity in m.3243A>G mitochondrial disease: the role of nuclear factors. Ann Clin Transl Neurol. (2018) 5:333–45. 10.1002/acn3.532 29560378 PMC5846390

[10] Seo GH Oh A Kim EN Lee Y Park J Kim T. Identification of extremely rare mitochondrial disorders by whole exome sequencing. J Hum Genet. (2019) 64:1117–25. 10.1038/s10038-019-0660-y 31451716

