# Supplementary material for: Case Report: Whole Exome Sequencing Identifies Compound Heterozygous Variants in TSFM Gene Causing Juvenile Hypertrophic Cardiomyopathy
Source: Front Cardiovasc Med. 2022 Jan 6;8:798985. doi: 10.3389/fcvm.2021.798985 (PMC8770926; doi:10.3389/fcvm.2021.798985)
Supplement: Supplementary file 2 [file Data_Sheet_1.pdf]

**SUPPLEMENTAL MATERIALS**

# Whole Exome Sequencing Identifies Compound Heterozygous Mutations in TSFM Gene Causing Juvenile Hypertrophic Cardiomyopathy.

Jamie O Yang[1], Hapet Shaybekyan[2,3], Yan Zhao[2,3], Xuedong Kang[2,3], Gregory A. Fishbein[4], Negar Khanlou[4], Juan C. Alejos[2], Nancy Halnon[2], Gary Satou[2], Reshma Biniwale[2], Hane Lee[4,5,*], Glen Van Arsdell[2], Stanley F. Nelson[2,4,5,7], Marlin Touma[1-3, 6-9], on behalf of The UCLA Clinical Genomics Center[1]& The UCLA Congenital Heart Defects-BioCore Faculty[1]

.

1. David Geffen School of Medicine, University of California Los Angeles, Los Angeles, CA.
2. Department of Pediatrics, David Geffen School of Medicine, University of California Los Angeles, Los Angeles, CA.
3. Neonatal/Congenital Heart Laboratory, Cardiovascular Research Laboratory, University of California Los Angeles, Los Angeles, CA.
4. Department of Pathology and Laboratory Medicine, David Geffen School of Medicine, University of California Los Angeles, Los Angeles, CA.
5. Department of Human Genetics, David Geffen School of Medicine, University of California Los Angeles, Los Angeles, CA.
6. Children’s Discovery and Innovation Institute, Department of Pediatrics, David Geffen School of Medicine, University of California Los Angeles, Los Angeles, CA.
7. Institute for Precision Health, David Geffen School of Medicine, University of California Los Angeles, Los Angeles, CA.
8. The Molecular Biology Institute, David Geffen School of Medicine, University of California Los Angeles, Los Angeles, CA.
9. Eli and Edythe Broad Stem Cell Research Center, David Geffen School of Medicine, University of California Los Angeles, Los Angeles, CA.

*Currently at 3billion, Inc, Seoul, South Kore

**Supplemental Video 1.** Echocardiography (apical view) depicts diminished cardiac function upon admission.

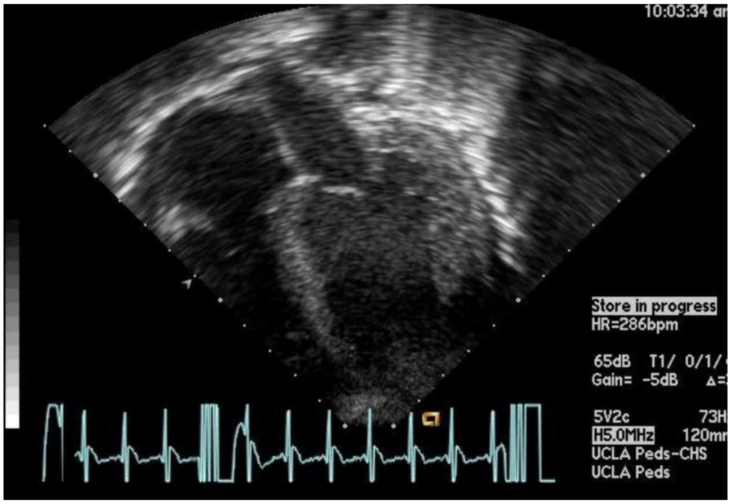


**Supplemental Figure 1.** Representative electrocardiogram recording of the proband upon admission at 3 years of age. High voltages indicate left ventricular hypertrophy.

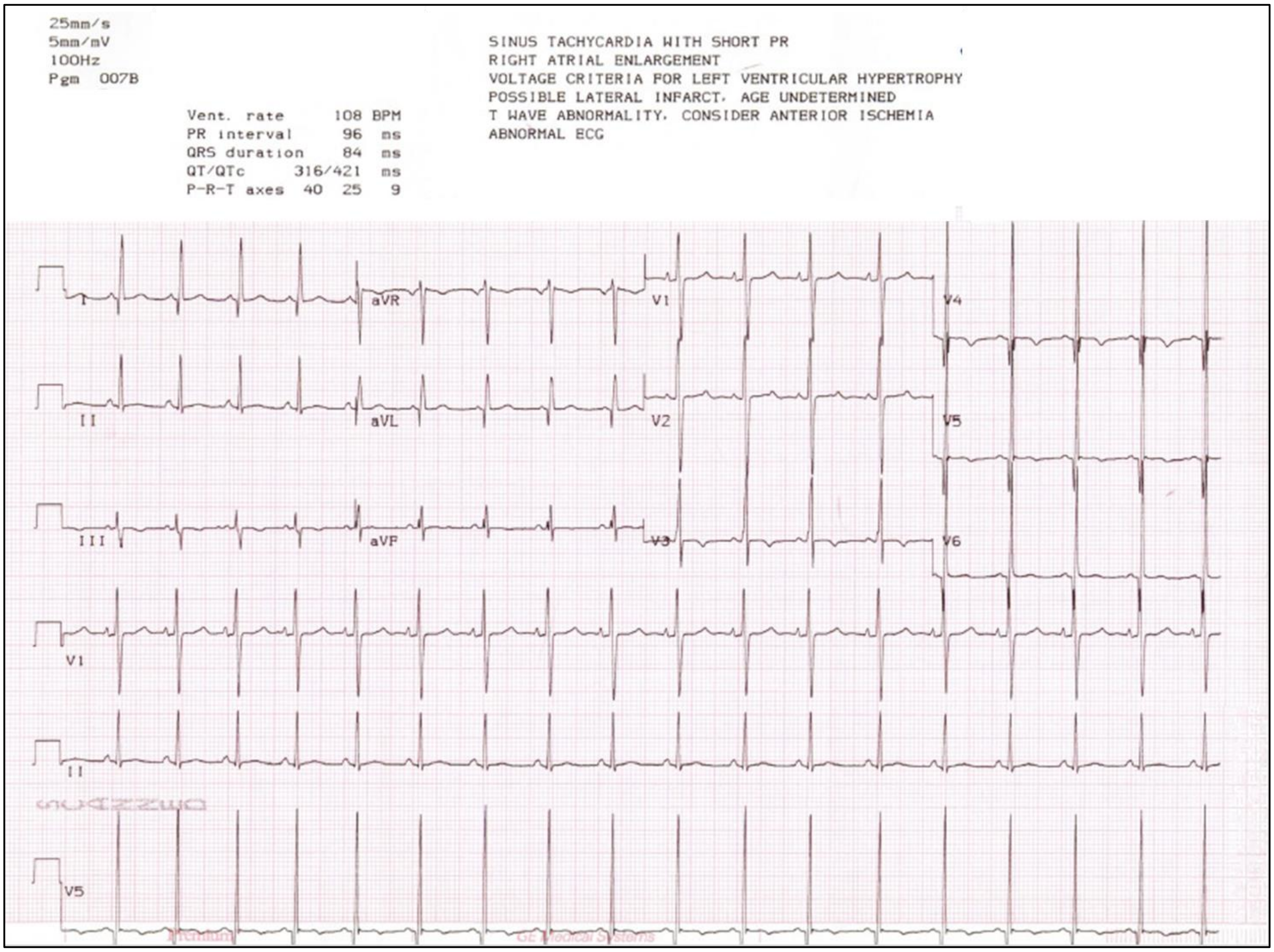

**Supplemental Table 1.** Biochemical Testing Results of the Proband

| Test | Result |
|---|---|
| Newborn Screen | Normal |
| Serum Lactate | Elevated |
| Plasma Amino Acid | Normal |
| Muscle Free/Total Creatinine | Normal |
| Muscle CPT1 and CPT Activities | Normal |
| Serum Acyl Carnitine | Normal |
| Acid Alpha Galactosidase Activity (GAA) | Negative |
| Urinary Hex4 Concentration | Normal |
| TORCH Titers | Negative |

**Supplemental Table 2. *TSFM* Mutations Reported to Date**

| Mutation(s) | Survival | Presence of Hypertrophic Cardiomyopathy | Clinical manifestations | | Serum Lactate | Reference |
|---|---|---|---|---|---|---|
| | | | Onset | Symptoms | | |
| c.997C>T [Arg333Trp] | 7 weeks | No | At birth | Hypotonia, generalized seizures, respiratory failure, encephalomyopathy, rhabdomyolysis | elevated | Smeitink 2006<br>PMID: 17033963 |
| c.997C>T [Arg333Trp] | 7 weeks | Yes | At 36 hours | Hypotonia, irregular breathing, hypertrophic cardiomyopathy | elevated | Smeitink 2006<br>PMID: 17033963 |
| c.997C>T [Arg333Trp] | Few months (no detail) | Yes | At birth<br>Exact onset not reported | hypotonia<br>Mitochondrial encephalomyopathy, hepatomegaly, hypertrophic cardiomyopathy | elevated | Smits 2011<br>PMID: 21119709 |
| c.934C>T [Arg312Trp]1 | 2 months | No | At birth<br>6 weeks | trunk hypotonia<br>liver insufficiency, tubulopathy | elevated | Vedrenne 2012<br>PMID: 21741925 |
| c.934C>T [Arg312Trp]1 | 2 weeks | Yes | At birth<br>At 2 days | trunk hypotonia, hypertrophic cardiomyopathy<br>generalized hypotonia, dystonic movements | elevated | Vedrenne 2012<br>PMID: 21741925 |
| c.997C>T [Arg333Trp] | 2 months | Yes | < 1 week | Hypotonia, Hypothermia, Hepatomegaly, hypertrophic cardiomyopathy | Not reported | Calvo 2012<br>PMID: 22277967 |
| c.997C>T [Arg333Trp] | 1 month | Yes | < 1 month | Hypertonia<br>Respiratory arrest<br>Hypertrophic cardiomyopathy | Not reported | Calvo 2012<br>PMID: 22277967 |
| c.944G>A [Cys315Tyr] / c.856C>T [Gln286X] | > 21 yrs | Yes | 10 months<br>7 years<br>9 years<br>Teenage<br>21 years | hypertrophic cardiomyopathy<br>cognitive decline<br>Leigh syndrome<br>optic atrophy<br>neuropathy, dystonia | elevated | Ahola 2014<br>PMID: 25037205 |
| c.944G>A [Cys315Tyr] / c.856C>T [Gln286X] (X = nonsense mutation) | > 15 yrs | Yes | 16 months<br>13 years<br>teenage | hypertrophic cardiomyopathy<br>neuropathy<br>optic atrophy | mildly elevated | Ahola 2014<br>PMID: 25037205 |
| c.856C>T [Gln286X] / c.106+4A>G [splice error] | > 21 yrs | No | 15 years<br>16 years | optic atrophy<br>tremor<br>peripheral neuropathy<br>ataxia | normal | Ahola 2014<br>PMID: 25037205 |
| c.944G>A [Cys315Tyr] | >21 yrs | No | 18 years<br>20 years | Hyperkinetic movement disorder<br>dystonia<br>optic atrophy<br>mild cognitive impairment | Mildly elevated | Traschutz 2019<br>PMID 30297209 |
| c.719G>C [Cys240Ser] | > 23 yrs | Yes | 6 years<br>11 years<br>Exact onset not reported | gross and fine motor clumsiness<br>hypertrophic cardiomyopathy, dystonia<br>optic atrophy, ataxia | normal | Emperador 2016<br>PMID 27677415 |
| c.408_409delGT [Leu137Glyfs*24] / c.505C>T [Leu169Phe] | >33 yrs | Dilated cardiomyopathy | Early childhood<br>27 | gross and fine motor clumsiness<br>mild muscle weakness<br>dilated cardiomyopathy | Mildly elevated | Perli 2019<br>PMID 30911037 |
| c.547G>A [Gly183Ser] | >8 yrs | Yes | 3 months<br>11 months<br>5 years<br>8 years | Hypotonia, lactic acidosis<br>hypertrophic cardiomyopathy<br>severe speech delay<br>sensorineural hearing loss | elevated | Scala 2019<br>PMID 31267352 |
| c.919C>T [Gln307*] / c.161G>A [Arg54Gln] * = nonsense variant | >15 yrs | No | At birth<br>2.5 years<br>5 years | Hypotonia<br>global developmental delay<br>hyperkinetic movement disorder<br>optic atrophy | elevated | Van Riesen 2021<br>PMID 33816677 |
| c.331_340delCAGGAAGGAA [Gln111ThrfsTer5] / c.57G>A [splice error (exon 5 skipping)] | >9 yrs | Yes | 3 months<br>4 months<br>6 months<br>1 year<br>5 years<br>9 years | Hepatomegaly, hypoglycemia, hypertriglyceridemia<br>lactic acidosis<br>hypertrophic cardiomyopathy<br>infantile spasm, truncal ataxia<br>optic atrophy<br>severe mental retardation | elevated | Go Hun Seo 2019<br>PMID 31451716 |
| | | | | | | |
| c.934C>T [Arg333Trp] / c.355G>C [Val119Leu] | >14 yrs | Yes | 3 years | hypertrophic cardiomyopathy | elevated | Our patient |

## Supplemental References (Correspond to Supplemental Table 2)


1. Smeitink, J. A., et al. (2006). "Distinct clinical phenotypes associated with a mutation in the mitochondrial translation elongation factor EFTs." Am J Hum Genet **79**(5): 869-877.
2. Smits, P., et al. (2011). "Mutation in subdomain G' of mitochondrial elongation factor G1 is associated with combined OXPHOS deficiency in fibroblasts but not in muscle." Eur J Hum Genet **19**(3): 275-279.
3. Vedrenne, V., et al. (2012). "Mutation in the mitochondrial translation elongation factor EFTs results in severe infantile liver failure." J Hepatol **56**(1): 294-297.
4. Calvo, S. E., et al. (2012). "Molecular diagnosis of infantile mitochondrial disease with targeted next-generation sequencing." Sci Transl Med **4**(118): 118ra110.
5. Ahola, S., et al. (2014). "Mitochondrial EFTs defects in juvenile-onset Leigh disease, ataxia, neuropathy, and optic atrophy." Neurology **83**(8): 743-751.
6. Traschutz, A., et al. (2019). "TSFM mutations cause a complex hyperkinetic movement disorder with strong relief by cannabinoids." Parkinsonism Relat Disord **60**: 176-178.

7. Emperador, S., et al. (2016). "Molecular-genetic characterization and rescue of a TSFM mutation causing childhood-onset ataxia and nonobstructive cardiomyopathy." Eur J Hum Genet **25**(1): 153-156.
8. Perli, E., et al. (2019). "Novel compound mutations in the mitochondrial translation elongation factor (TSFM) gene cause severe cardiomyopathy with myocardial fibro-adipose replacement." Sci Rep **9**(1): 5108.
9. Scala, M., et al. (2019). "Novel homozygous TSFM pathogenic variant associated with encephalocardiomyopathy with sensorineural hearing loss and peculiar neuroradiologic findings." Neurogenetics **20**(3): 165-172.
10. van Riesen, A. K., et al. (2021). "Novel Mutation in the TSFM Gene Causes an Early-Onset Complex Chorea without Basal Ganglia Lesions." Mov Disord Clin Pract **8**(3): 453-455.
11. Seo, G. H., et al. (2019). "Identification of extremely rare mitochondrial disorders by whole exome sequencing." J Hum Genet **64**(11): 1117-1125.
